# Activity of TREK-2-like Channels in the Pyramidal Neurons of Rat Medial Prefrontal Cortex Depends on Cytoplasmic Calcium

**DOI:** 10.3390/biology10111119

**Published:** 2021-10-30

**Authors:** Beata Dworakowska, Maciej Gawlak, Ewa Nurowska

**Affiliations:** 1 Department of Physics and Biophysics, Institute of Biology, Warsaw University of Life Sciences, 02-776 Warsaw, Poland; beata_dworakowska@sggw.edu.pl; 2 Laboratory of Physiology and Pathophysiology, Centre for Preclinical Research and Technology, Faculty of Pharmacy, Medical University of Warsaw, 02-097 Warsaw, Poland; maciej.gawlak@wum.edu.pl

**Keywords:** TREK-2 channel, calcium signaling, PI(4,5)P2 signaling

## Abstract

**Simple Summary:**

The pyramidal neurons of rat prefrontal cortex express potassium channels identified as a non-canonical splice variant of the TREK-2 channel. The main function of TREK channels is to regulate the resting membrane potential. We showed that cytoplasmic Ca^2+^ upregulates the activity of TREK-2-like channels. Previous studies have indicated that the activation of TREK-2 channels is mediated by PI(4,5)P2, a polyanionic lipid in the inner leaflet of the plasma membrane. While TREK channels are believed to not be regulated by calcium, our work shows otherwise. We propose a model in which calcium ions enable the formation of PI(4,5)P2 nanoclusters, which stabilize active conformation of the channel.

**Abstract:**

TREK-2-like channels in the pyramidal neurons of rat prefrontal cortex are characterized by a wide range of spontaneous activity—from very low to very high—independent of the membrane potential and the stimuli that are known to activate TREK-2 channels, such as temperature or membrane stretching. The aim of this study was to discover what factors are involved in high levels of TREK-2-like channel activity in these cells. Our research focused on the PI(4,5)P2-dependent mechanism of channel activity. Single-channel patch clamp recordings were performed on freshly dissociated pyramidal neurons of rat prefrontal cortexes in both the cell-attached and inside-out configurations. To evaluate the role of endogenous stimulants, the activity of the channels was recorded in the presence of a PI(4,5)P2 analogue (PI(4,5)P2DiC8) and Ca^2+^. Our research revealed that calcium ions are an important factor affecting TREK-2-like channel activity and kinetics. The observation that calcium participates in the activation of TREK-2-like channels is a new finding. We showed that PI(4,5)P2-dependent TREK-2 activity occurs when the conditions for PI(4,5)P2/Ca^2+^ nanocluster formation are met. We present a possible model explaining the mechanism of calcium action.

## 1. Introduction

The human prefrontal cortex is involved in a variety of cognitive functions [1]. The prefrontal cortex can be divided into two regions: the medial prefrontal cortex (mPFC) and the lateral prefrontal cortex [2]. Medial prefrontal cortex lesions leading to cognitive impairment have been associated with various human brain disorders, such as depression, anxiety disorders, schizophrenia, autism spectrum disorders, Alzheimer’s disease, Parkinson’s disease, and addiction [3]. Some of these pathological processes may involve two-pore domain potassium channels (K2P), which are thought to play a significant role in neuronal excitability and in the resting membrane potential [4]. 

TREK-2 belongs to the TREK subfamily of K2P channels, together with TREK-1 and TRAAK. TREK-1 and TREK-2 channels share similar properties [5]. TREK channels are involved in depression, neuroprotection, pain, and anaesthesia [6,7,8,9,10,11,12,13]. Mice in which the TREK-1 channel has been deleted have an increased vulnerability to both epileptic seizure and cerebral ischemia [14] and are resistant to depression [6,15]. TREK channels show relatively low basal activity that is increased by physical and chemical stimuli, including temperature, membrane stretch, pH, unsaturated fatty acids [16,17,18,19,20,21,22,23,24,25], and PI(4,5)P2 [26]. The C-terminus of TREK is crucial for the sensitivity to intracellular pH and membrane stretching [27,28] as well as PI(4,5)P2, the phosphatidylinositol present in the inner leaflet of the plasma membrane. The TREK C-terminus contains several positively charged amino acids that are electrostatically attracted to the negative charges of the PI(4,5)P2 anionic head group, which causes TREK to enter an active state [26,29,30]. The protonation of glutamic acid in the C-terminus at low pH enhances the cluster of positive charges in the C-terminus and tightens the electrostatic interaction with PI(4,5)P2. Polycationic molecules such as poly-L-lysine disturb that interaction, and in their presence, the TREK channel remains in an inactive state insensitive to low pH and less sensitive to membrane stretch [26]. The preservation of mechanosensitivity in these experiments indicates that other factors than PI(4,5)P2 play a role in regulation by mechanical stimuli [31]. TREK channels are downregulated by the activation of G-coupled receptors activating phospholipase C, which hydrolyses PI(4,5)P2 to diacylglycerol (DAG) and inositol triphosphate (IP3), with the latter releasing Ca^2+^ from internal stores. The depletion of PI(4,5)P2 caused by hydrolysis and the resulting loss of membrane interaction of the C-terminus inhibits TREK channels [30,32,33]. TREK channels are also inhibited by the phosphorylation of multiple residues on the cytoplasmic C-terminus domain by protein kinase A and C following the stimulation of G-coupled receptors [12,34,35,36]. Phosphorylation adds negative charges to the C-terminus domain, which may reduce binding to the plasma membrane and, therefore, the channel activity [18,37]. 

The role of PI(4,5)P2 in the gating of TREK channels is more complicated than just the stabilization of an active conformation by tethering the C-terminal domain to the plasma membrane, as the inhibition of TREK currents by PI(4,5)P2 was also observed. This inhibition occurred at higher concentrations of PI(4,5)P2 and was eliminated by pre-treatment with poly-L-lysine [38]. Site-directed mutagenesis and deletion studies have identified residues in the C-terminus responsible for activating and inhibiting the action of PI(4,5)P2 [26,38]. It is suggested that ATP is another factor that reduces TREK activity by increasing the PI(4,5)P2 pool in the plasma membrane. The increase in channel activity in whole-cell recordings was observed when the pipette solution was free of ATP or when the patches were excised into an ATP-free bath solution. Exogenous PI(4,5)P2 inhibited the spontaneous increase in TREK currents induced by ATP depletion. ATP inhibition of TREK channels can be mediated by phosphatidylinositol kinase, which increases the amount of PI(4,5)P2 in the plasma membrane, and the increase in PI(4,5)P2 has an inhibitory effect on TREK currents [21,39,40,41]. This inhibitory effect of PI(4,5)P2 can be relieved by anionic phospholipids, such as phosphatidic acid, which is a TREK agonist. An indirect stimulatory effect of PI(4,5)P2 on TREK currents may involve the production of phosphatidic acid by phospholipase D [42].

Potassium channels identified as a non-canonical splice variant of TREK-2 are expressed in pyramidal neurons of the medial prefrontal cortex of rats [36]. TREK-2-like channels were often spontaneously active in these cells during patch-clamp recordings, though no chemical or physical stimuli were applied to the channels. To further analyse the mechanisms controlling the activity of these channels, we studied the PI(4,5)P2-dependence of TREK-2-like channel activation. Our experiments showed that cytoplasmic Ca^2+^ plays a role in TREK-2 regulation by PI(4,5)P2. 

## 2. Materials and Methods

The experimental procedures used in this study conform to institutional and international guidelines for the ethical use of animals in accordance with Directive 2010/63/EU of The European Parliament and of The Council of 22 September 2010 on the protection of animals used for scientific purposes. The methods used for sacrificing the animals were in accordance with Annex IV of the Directive. The experiments were performed on young, 20-day-old, male Wistar rats (WAG Cmd) obtained from a local animal house. 

### 2.1. Immunofluorescence Staining

The anesthetized rats (sodium pentobarbital, 75 mg/kg, i.p.) were perfused with phosphate-buffered saline (PBS) and 4% paraformaldehyde in PBS (PFA). The brains were post fixed in PFA (4 °C) overnight, impregnated with sucrose (30% *w*/*v* in PBS, 4 °C), rapidly frozen in dry-ice-cold heptane, cut in cryotome (40 μm), and embedded and kept (−20 °C) in a cryoprotectant (30% glycerol, 30% ethylene glycol, and 0.02M PBS). For fluorescent staining, the cryoprotectant was washed with PBS and the brain slices were blocked in 5% normal goat serum in PBS with 0.01% Triton X-100 (NGST) and immersed overnight (4 °C) in rabbit anti-TREK2 primary antibody (Invitrogen, PA5-77608) solution in NGST. Next, the slices were washed with PBS with 0.01% Triton X-100 (PBST) and incubated overnight (4 °C) with goat anti-rabbit Alexa 568-conjugated secondary antibody (Invitrogen, A11011) solution in NGST. Furthermore, after washing, an analogical procedure was applied with a next pair of antibodies, i.e., mouse anti-NeuN primary antibody (Millipore, MAB377) and goat Alexa 488-conjugated anti-mouse secondary antibody (Invitrogen, A11001). Next, the slices were washed with PBST and mounted in Vectashield with a DAPI medium (Vector, catalogue number H-1200) [43]. Parallelly, the cerebellum tissue was processed in the same manner. The pattern of the signal in the cerebellum cortex, namely in the granular and molecular layers, was used to verify the specificity of the anti-TREK2 antibody. Signal of fluorescence was measured with Olympus Fluoview FV1000 confocal laser-scanning microscope and analysed with Fiji software [44]. The figures were prepared with Fiji and Office software.

### 2.2. Electrophysiological Recordings

All experiments were performed on freshly isolated and enzymatically dispersed pyramidal neurons of mPFC. The preparation of the mPFC slices was performed as previously published [36]. The animals were decapitated (under ethyl chloride anaesthesia), and the brains were removed and placed in a cold (1–4 °C) solution containing NaCl (125 mM), NaHCO_3_ (25 mM), KCl (3 mM), NaH_2_PO_4_ (1.25 mM), CaCl_2_ (2 mM), MgCl_2_ (1 mM), and glucose (25 mM). The solution was saturated with 95% oxygen and 5% CO_2_. The pH was adjusted to 7.4 with NMDG. The osmolality was 330 mOsm. Coronal slices of cerebral prefrontal tissue (300 μm thick) were cut with a vibratome (Leica VT1200S) and stored at room temperature (21–22 °C) in bubbled (95% O_2_ and 5% CO_2_) solution containing NaCl (125 mM), NaHCO_3_ (25 mM), KCl (3 mM), NaH_2_PO_4_ (1.25 mM), CaCl_2_ (0.5 mM), MgCl_2_ (6 mM), and glucose (25 mM). The pH was adjusted to 7.4 with NMDG. The osmolality was 330 mOsm. A fragment of a slice corresponding to mPFC was cut with scissors and kept in a solution containing sodium isethionate (140 mM), HEPES (15 mM), KCl (2 mM), MgCl_2_ (4 mM), glucose (20 mM), CaCl_2_ (0.1 mM), and 0.75 mg/mL protease type XIV (Sigma-Aldrich, the pH of the solution was adjusted to 7.4 with NMDG; osmolality was 320 mOsm) for 10–20 min at 32–33 °C. Enzymatically treated slices were treated for 30 min in the same solution lacking protease but with addition of H89 (10 μM, Tocris) to block protein kinase A. After H89 pre-treatment, the tissue fragments were dispersed using Pasteur pipettes and transferred to a recording chamber (RC-27 N, Warner Instr., Holliston, MA, USA), coated with Poly-L-lysine hydrobromide (50 μg/mL). The identification of pyramidal neurons was performed optically on the basis of the size and shape of the cell body and the presence of apical dendrites. 

Patch-clamp recordings of the single-channel currents were performed in the cell-attached or inside-out configurations at room temperature. A low-pass Bessel filter of 5 kHz was applied during the recordings. The data were digitized, added onto a computer, with sampling frequencies of 10 or 100 kHz. The extracellular control solution contained KCl (130 mM); EGTA (0.3 mM); HEPES (10 mM); glucose (10 mM); CaCl_2_ (0–2 mM); and in some experiments, paxilline (1 μM, Sigma-Aldrich, Poznan, Poland). The control solution was then exchanged for the experimental solution containing the tested substances: arachidonic acid (Sigma-Aldrich), PIP2DiC8 (Echelon Biosciences, Salt Lake City, UT, USA), neomycin (Sigma-Aldrich), m-3M3FBS (EMD Millipore Corp. Darmstadt, Germany), and thapsigargin (Tocris, Bio-Techne, Warsaw, Poland). The tested substance was applied by changing the bath solution, i.e., by changing the contents of the entire recording chamber (~0.6 mL), which lasted from about 1 min to about 2 min. The rate of solution exchange was about 1 mL/minute. The concentration of Ca^2+^ in these experimental solutions was as follows: with arachidonic acid, 0 Ca^2+^; PIP2DiC8, 0 or 0.1 mM Ca^2+^; m-3M3FBS, 0.1 or 1.7 mM Ca^2+^; and thapsigargin, 1.7 mM Ca^2+^. The concentration of Ca^2+^ was estimated based on the concentration of EGTA and CaCl_2_. The control solutions for these experiments contained corresponding amounts of Ca^2+^. The pipette solution contained K acetate (125 mM), KCl (5 mM), HEPES (10 mM), CaCl_2_ (2 mM), LaCl_3_ (2.5 μM), penitrem A (15 nM, Tocris), and charybdotoxin (100 nM, Alomone Labs, Jerusalem, Israel ), and the last two compounds were added to the block BK channel. A junction potential of 6 mV was not corrected during the experiments.

In some experiments, the cells were pre-treated with BAPTA-AM for about 30 min. 

P_open_ corresponding to the probability of opening all channels in the patch was evaluated with Clampfit (pClamp 10.6, Molecular Devices, San Jose, CA, USA). The calculation for the probability did not take the number of ion channels into consideration. The threshold of the opening transition was set at one-half of the unitary current. Open dwell times were calculated by fitting the probability density function (the sum of two exponential terms [45]) to open time histograms with the use of Clampfit. *τ_oi_* corresponds to the time constant of the *i*th component, *P* corresponds to its normalized area. Openings shorter than 50 μs were not included in the histograms. All results are shown as means ± SEM. Nonparametric tests (unpaired Mann–Whitney test or paired Wilcoxon test) or the *t*-test with or without Welch’s correction were used to make comparisons between the two groups. To compare the means of three groups, we used one-way ANOVA followed by multiple comparisons tests: Sidak’s or Bonferroni’s (parametric test), or Dunn’s (nonparametric test). Statistical analyses were performed with GraphPad Prism 7.00. 

## 3. Results

### 3.1. Role of Calcium in TREK-2-Like Channel Activation

**Figure 1:**
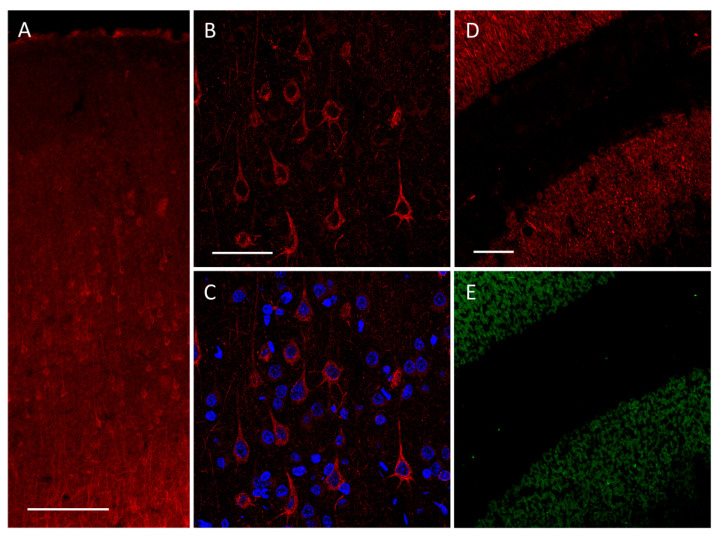
Immunofluorescent staining of TREK-2 performed on PFC. (**A**) The most prominent signal of potassium channel staining is localized in neurons, particularly in the V layer of PFC. (**B**) With higher magnification, the morphology of Trek2-positive neurons is evident: the shape of cells marked by a TREK-2 signal (red) is typical for pyramidal neurons. Trek2 is localized in the soma (and in protrusions). (**C**) DAPI (blue) staining reveals neuronal and non-neuronal cell nuclei. (**D**) Control staining with immunosignal of TREK-2 and (**E**) immunosignal of NeuN in the cerebellum cortex. The latter is a marker of granular cells. The TREK-2 signal is localized in the granular layer with a molecular layer deprived of TREK-2 signals. Such a distribution stays in line with reports on KCNK10 transcript localization and with electrophysiological data [47,48]. Scales bars: (**A**) 200 µm, (**B**,**C**) 50 µm, and (**D**,**E**) 100 µm.

We already showed that pyramidal neurons in the medial prefrontal cortex of rats express high-conductance TREK-2-like channels [36,46]. The presence of TREK-2 channels in pyramidal neurons was confirmed by immunofluorescent staining of PFC slices (Figure 1). 

**Figure 2:**
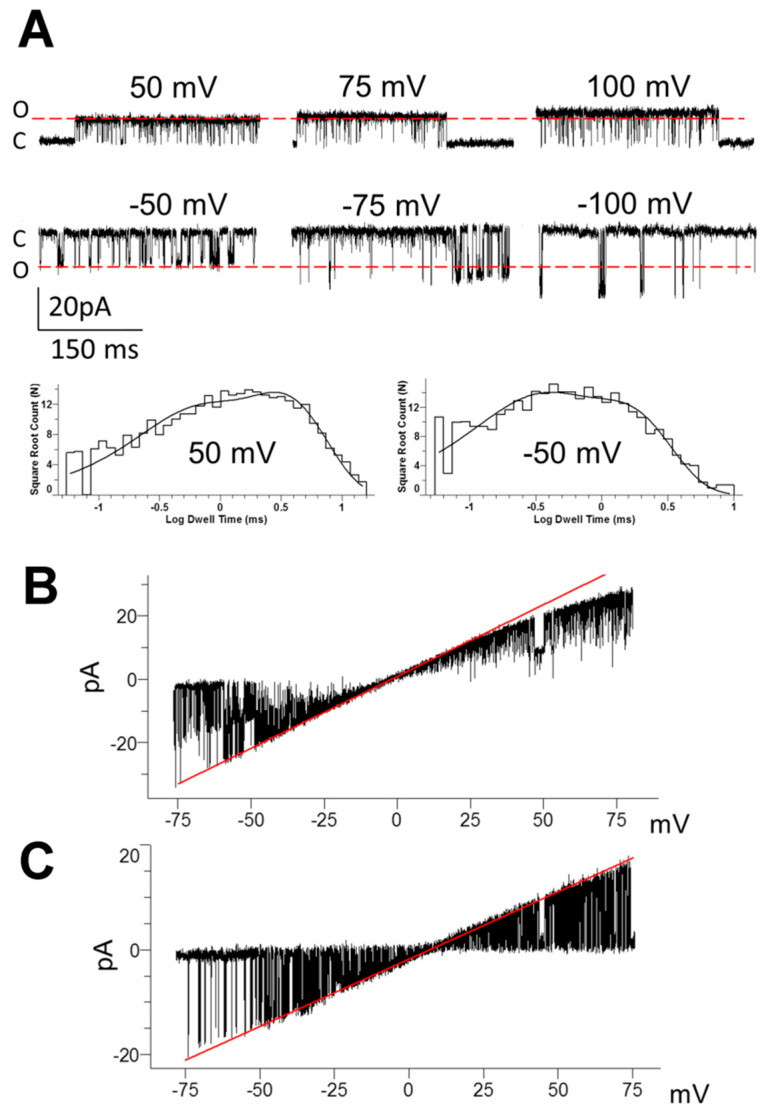
Single-channel currents of TREK-2-like channels in the mPFC pyramidal neurons. (**A**) Original recordings of currents at different patch membrane potentials in the cell-attached configuration. Red lines, which represent the unitary amplitude at 50 mV (upper traces) or -50 mV (lower traces), are shown to emphasize the rectifying properties of the channel; C—closed channel, O—open channel current; traces were recorded with the low-pass Bessel filter of 5kHz; and the pipette solution contained no divalent cations. Histograms of open dwell times for the given potentials fitted with the probability density function are shown below the traces; 50 mV: *τ_o1_* = 0.42 ms (*p* = 0.32), *τ_o2_* = 2.83 ms (*p* = 0.68); −50 mV: *τ_o1_* = 0.22 ms (*p* = 0.38), *τ_o2_* = 1.28 ms (*p* = 0.62) (**B**) TREK-2-like channel activity recorded during ramp membrane depolarization from −75 mV to 75 mV in the cell-attached configuration. The red line corresponds to the conductance of two channels at 453 pS (half of which represents single channel conductance equal to 226 pS) and reveals the presence of inward rectification. The pipette solution contained no divalent cations. (**C**) TREK-2-like channel activity recorded during ramp membrane depolarization from −75 mV to 75 mV in the inside-out configuration. The red line corresponds to a single-channel conductance of 253 pS; no rectification is visible. Bath solutions: 0.1 mM Ca^2+^ and 0 mM Mg^2+^; pipette solution: 2 mM Ca^2+^ and 2 mM Mg^2+^. In all recordings, the bath and pipette solutions contained 130 mM K^+^ and the bath solution contained 0.1 mM Ca^2+^.

Patch clamp experiments were performed on freshly dissociated pyramidal neurons from rat mPFC in ‘‘symmetrical” K^+^ solutions. In such conditions, the cell membrane potential was close to 0 mV. In the cell-attached configuration, high-conductance potassium currents were observed in hyperpolarized and depolarized potentials (Figure 2A). In the cell-attached configuration in the presence of 1.7 mM Ca^2+^ in the extracellular bath solution, P_open_ was 0.989 ± 0.126 (*n* = 36) at 50 mV and 0.521 ± 0.087 (*n* = 28) at -50 mV (cells without TREK-2 like channel activity were excluded from the statistics). The channel was previously identified in our laboratory as a non-canonical splice variant of the TREK-2-like channel (first described by Gu et al. 2002) based on high conductance, weak rectification properties, membrane stretch, and temperature sensitivity and the inhibition by ruthenium red [36,47]. We confirmed that conductance depended on the membrane potential and magnesium presence in the pipette solution. In the cell-attached configuration, Mg^2+^ present in the pipette solution reduced inward conductance from 221 ± 10 pS (*n* = 13, 0 mM Mg^2+^) to 179 ± 7 pS (*n* = 23, 2 mM Mg^2+^). The outward conductance decreased with increasing depolarization, but its value did not exceed that for inward currents. Figure 2A,B show weak rectifying properties of the TREK-2-like channel. In excised patches in the absence of Mg^2+^ in the bath solution, rectification was abolished (Figure 2C). The duration of open events depended on the membrane potential, as open time constants changed from 0.353 ± 0.038 ms and 1.506 ± 0.135 ms at −50 mV to 0.901 ± 0.241 ms and 4.844 ± 1.02 ms at 50 mV (cell-attached configuration, *n* = 7, Figure 2A). 

**Figure 3:**
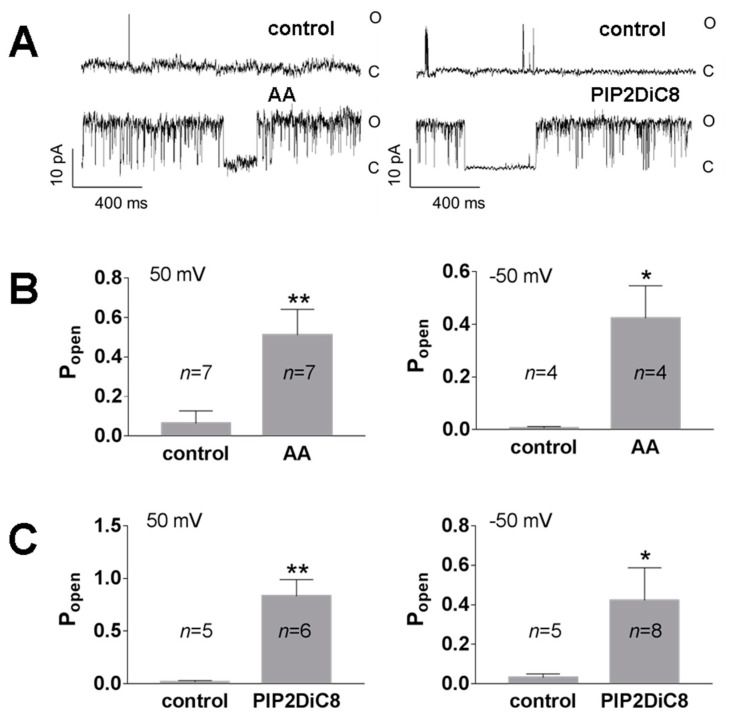
TREK-2 channel activators AA and PI(4,5)P2DiC8 induced an increase in P_open_ in excised patches with low spontaneous activity. (**A**) Original recordings at the control and experimental conditions at 50 mV; the left traces represent the activity of a TREK-2-like channel in the control solution (0 mM Ca^2+^) and after the change to the solution containing AA (0 mM Ca^2+^, 10 μM AA); and the right traces represent the experiment in which the control solution (0.1 mM Ca^2+^) was exchanged for the solution with PIP2DiC8 (0.1 mM Ca^2+^, 10 μM PI(4,5)P2DiC8). Low-pass filters were set at 0.5 kHz. (**B**) Mean P_open_ for the experiments with 10 μM AA at two membrane potentials: 50 mV and −50 mV; ** *p* = 0.009, * *p* = 0.015, unpaired *t* test. (**C**) Mean P_open_ for experiments with 10 μM PI(4,5)P2DiC8 at two membrane potentials: 50 mV and −50 mV; ** *p* = 0.004, * *p* = 0.041, Mann–Whitney test.

**Figure 4:**
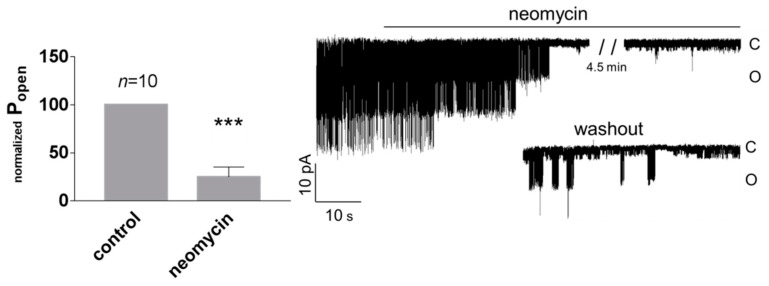
TREK-2-like channels are inhibited by polycationic neomycin (15 μg/mL) in excised patches. The control solution was exchanged for the solution with neomycin. Both solutions contained 0.1 mM Ca ^2+^. P_open_ was normalized in comparison with P_open_ in the control solution, which was set at 100%. P_open_ for recordings at 50 mV and −50 mV was pooled together. *** *p* < 0.002, one sample t-test. Original single-channel recordings at −50 mV at the following conditions: control, neomycin, and washout. The traces were filtered with a low-pass filter set at 1 kHz.

In many published studies, the high activity of TREK channels had to be induced by activating agents, whereas spontaneous activity of native TREK-2 channels was rarely reported [49]. Under our experimental conditions, high spontaneous activity of TREK-2-like channels was often observed. The spontaneous activity was observed with or without H89 pre-treatment. H89 was used to exclude the inhibitory effect of channel phosphorylation by kinase A [36]. TREK-2 is a thermo- and mechanosensitive channel controlled by various intracellular signalling pathways, including arachidonic acid (AA) and PI(4,5)P2. We confirmed on the excised patches that the activity of TREK-2-like channels was enhanced by the application of AA (10 μM) and PI(4,5)P2DiC8 (10 μM, a water-soluble analogue of PI(4,5)P2) to the cellular side of the patch (Figure 3). Ion channels dependent on PI(4,5)P2 are inhibited by polycationic agents (e.g., neomycin), which act by screening the negative charges of PI(4,5)P2 [26,50,51]. We confirmed that neomycin (15 μg/mL) decreased the activity of TREK-2-like channels (Figure 4) when applied to the cytoplasmic side of the membrane in excised patches. 

**Figure 5:**
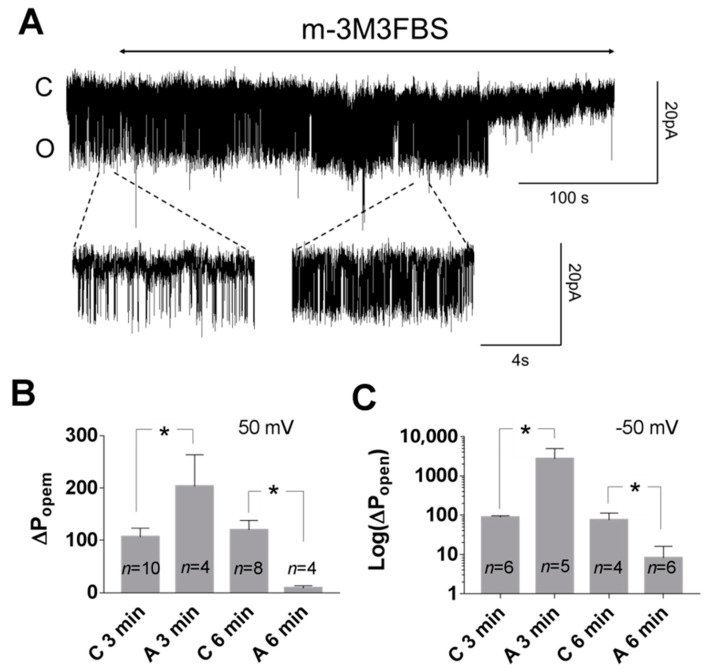
Effect of the PLC activator (m-3M3FBS) on TREK-2 like channel activity in the cell-attached configuration. (**A**) Original trace showing the action of the PLC activator (25 μM) on the activity of the TREK-2-like channel at −50 mV. A temporary increase in the activity after about 3 min of m-3M3FBS administration is visible in the right expanded fragment of the upper trace. (**B,C**) The columns represent normalized P_open_ after 3 minutes in the control solution (C_3min_) or the PLC activator (A_3min_) and after 6 minutes in the control solution (C_6min_) or in the PLC activator (A_6min_): 100% activity was attributed to the P_open_ at the beginning of recording in the control solution (P_open_ at 0 min). C_3min_ and A_3min_ represents $\frac{\mathrm{Popen}\;\mathrm{at}\;3\;\min}{\mathrm{Popen}\;\mathrm{at}\;0\;\min}*100\%$, and C_6min_ and A_6min_ represent $\frac{\mathrm{Popen}\;\mathrm{at}\;6\;\min}{\mathrm{Popen}\;\mathrm{at}\;0\;\min}*100\%$ in the control solution or in the presence of the PLC activator, respectively: (**B**) 50 mV, C_3min_ vs. A_3min_ * *p* = 0.039, C_6min_ vs. A_6min_ * *p* = 0.033; Bonferroni’s multiple comparison test; (**C**) −50 mV, C_3min_ vs. A_3min_ * *p* = 0.045, C_6min_ vs. A_6min_ * *p* = 0.048, Mann–Whitney test. In all experiments, the bath solution contained Ca^2+^ (0.1 or 1.7 mM).

**Figure 6:**
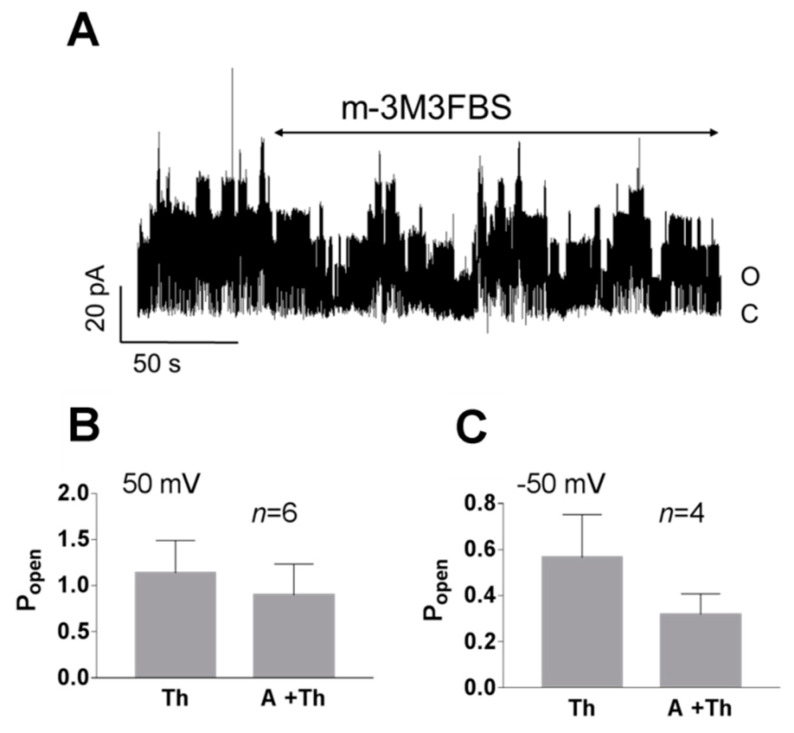
Effect of the 3-minute application of a PLC activator (m-3M3FBS) in the presence of thapsigargin in the cell-attached configuration. (**A**) Original recording presenting TREK-2-like channel activity in the pyramidal neuron at 50 mV pre-treated with thapsigargin (Th, 1 μM); the PLC activator (m-3M3FBS, 25 μM) was applied for 3 min in the presence of Th (A+Th) and Ca^2+^ (1.7 mM) in the bath solution. (**B**,**C**) No significant change in the P_open_ occurred when thapsigargin was administered to the cell for 20–30 min prior to the application of m-3M3FBS: (**B**) 50 mV, *p* = 0.563; (**C**) −50 mV, *p* = 0.375; Wilcoxon matched-pairs test. The bath solution contained 1.7 mM Ca^2+^.

**Figure 7:**
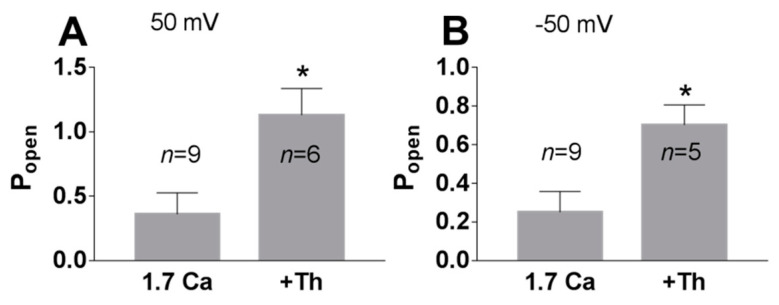
Effect of thapsigargin on the TREK-2 like channel activity in the cell-attached configuration. The cells were perfused with a bath solution containing 1.7 mM Ca^2+^ with or without 1 μM thapsigargin (20–30 min pre-treatment). A significant difference in the TREK-2 like channel P_open_ was observed: (**A**) 50 mV, * *p* = 0.025, Mann–Whitney test; (**B**) −50 mV, * *p* = 0.023; Mann–Whitney test.

Phospholipase C cleaves PI(4,5)P2 to produce second messengers: IP3 and DAG. To further test whether the PI(4,5)P2-dependent pathway is involved in TREK-2-like channel activity, we studied the effect of a phospholipase C activator m-3M3FBS. M-3M3FBS decreases the PI(4,5)P2 pool and activity of ion channels dependent on PI(4,5)P2 within a few hundred seconds [52,53]. We evaluated the changes in channel activity (P_open_) over time in the presence of m-3M3FBS in a bath solution and compared them with changes in the activity in control conditions. P_open_ in the control solution remained approximately unchanged during the recording period (Figure 5), whereas the application of m-3M3FBS resulted in bimodal change in the channel activity. After ~3 min in the presence of m-3M3FBS, P_open_ increased, and then, after another period of ~3 min, the activity of the channels significantly diminished. m-3M3FBS was shown to induce the release of Ca^2+^ from intracellular stores [54]. When m-3M3FBS was used in the presence of thapsigargin (Th), no initial increase in P_open_ was observed (Figure 6). Thapsigargin blocks the ability of the cell to pump calcium into the endoplasmic reticula, which results in elevated cytosolic calcium and emptying of intracellular calcium stores. We observed that thapsigargin alone significantly increased the activity of TREK-2-like channels (P_open_) when applied at high extracellular Ca^2+^ concentrations (Figure 7). We concluded that the initial increase in P_open_ induced by the application of m-3M3FBS is due to the increased level of cytoplasmic calcium, while the subsequent decrease in P_open_ is due to the depletion of PI(4,5)P2. Therefore, we performed experiments to test the possibility that calcium increases the activity of TREK-2-like channels.

**Figure 8:**
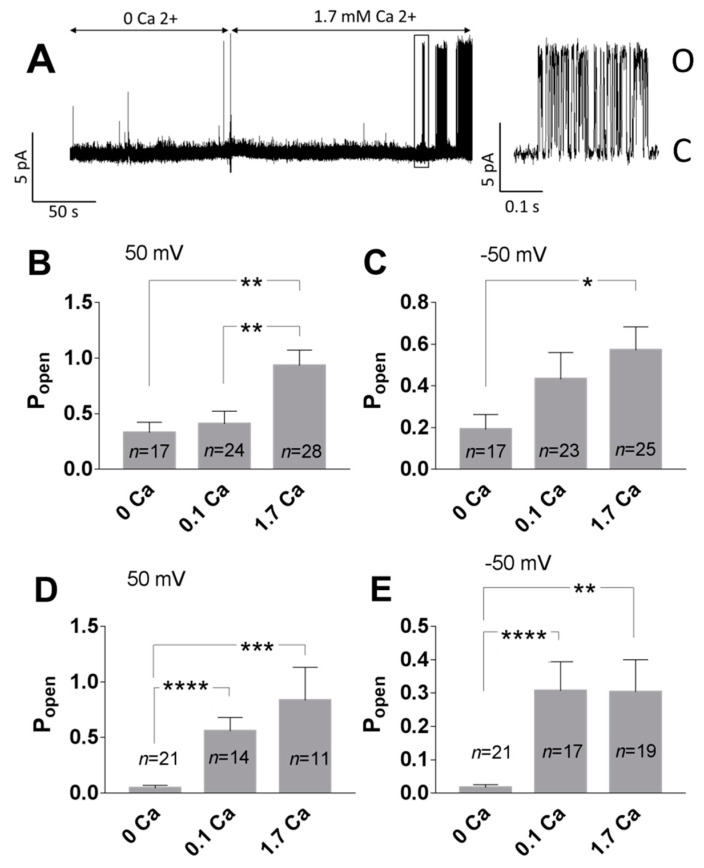
Ca^2+^ increased the activity of TREK-2-like channels. (**A**) Example of the single-channel recording performed in the inside-out configuration. The bath solution without Ca^2+^ was exchanged for a solution with 1.7 mM Ca^2+^. After 2–4 min of perfusion with Ca^2+^, the activity of the TREK-2-like channel was observed. The fragment indicated with the rectangle was elongated to show single openings of the channel. A low-pass filter of 0.5 kHz was applied. (**B**,**C**) P_open_ in the cell-attached experiments; experiments with no TREK-2-like channel activity were excluded. Ca^2+^ was applied in the extracellular bath solution. (**B**) 50 mV, ** *p* < 0.0085, Sidak’s multiple comparisons test; (**C**) −50 mV, * *p* = 0.0495, Sidak’s multiple comparisons test. (**D**,**E**) P_open_ in the inside-out experiments. Ca^2+^ was applied to the cytoplasmic side of the membrane. (**D**) 50 mV, *** *p* = 0.0002, **** *p <* 0.0001, Dunn’s multiple comparisons test; (**E**) −50 mV, **** *p* < 0.0001, ** *p* = 0.0017, Dunn’s multiple comparisons test.

The impact of Ca^2+^ on the TREK-2 like channel activity was tested in the cell-attached and inside-out configurations. In the cell-attached configuration, Ca^2+^ applied extracellularly in the bath solution increased the P_open_ of TREK-2-like channels compared with experiments performed in the absence of Ca^2+^. The increase was statistically significant at 1.7 mM Ca^2+^ (Figure 8B,C). A bath solution cannot directly affect the ion channel in the recorded patch in the cell-attached configuration, so the high activity of the TREK-2-like channel was a consequence of cytoplasmic factors. To test whether the effect depends on the intracellular calcium level, we measured the channel activity in the inside-out configuration. With 0 mM Ca^2+^, the TREK-2 like channels had low activity (Figure 8A,D,E). The application of Ca^2+^ to the cytoplasmic side of the plasma membrane (0.1 mM or 1.7 mM) resulted in a significant increase in the mean P_open_ (Figure 8A,D,E). 

**Figure 9:**
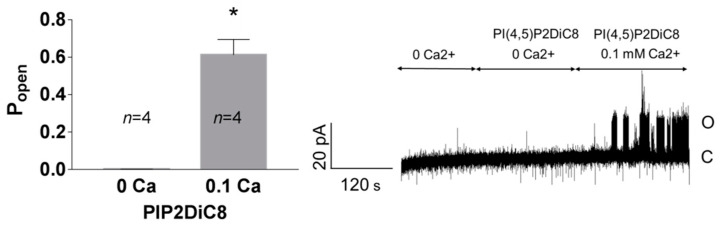
The effect of PI(4,5)P2DiC8 on P_open_ at 50 mV depends on Ca^2+^. In the inside-out patches, the bath solution containing 10 μM PI(4,5)P2DiC8 with no free calcium ions was exchanged for the solution containing PI(4,5)P2DiC8 and 0.1 mM Ca^2+^. Trace represents the exemplary experiment. * *p* = 0.029, Mann–Whitney test.

Enhancement of the activity of TREK-2-like channels by PI(4,5)P2 required the presence of Ca^2+^ at the intracellular side of the plasma membrane (Figure 9). In the experiments with no Ca^2+^, the activity of the recorded channels remained at very low levels when the inside-out patches were perfused with 10 μM PI(4,5)P2DiC8. The activity increased significantly when the 0.1 mM Ca^2+^ was added to the bath solution. 

### 3.2. Pattern of TREK-2-Like Channel Activity

**Figure 10:**
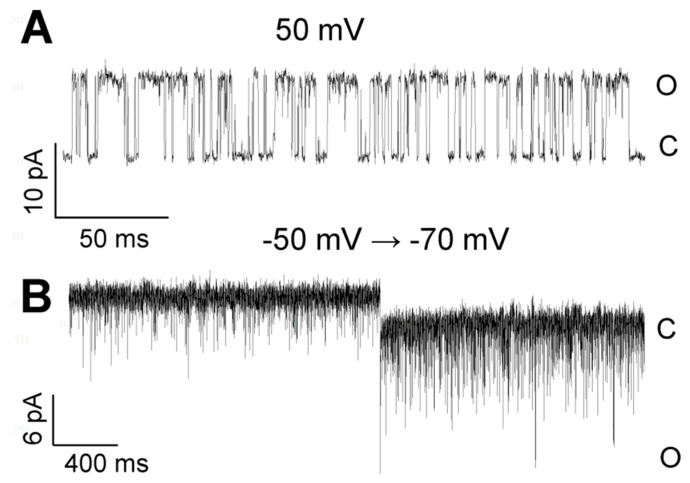
Examples of TREK-2-like channel activity in two different modes in the cell-attached configuration. (**A**) Burst of a well-resolved openings at 50 mV; a low-pass filter of 5 kHz. (**B**) The flickering mode: the potential changed from −50 mV to −70 mV; a low-pass filter of 5 kHz. The bath solution contained 1.7 mM Ca^2+^.

**Figure 11:**
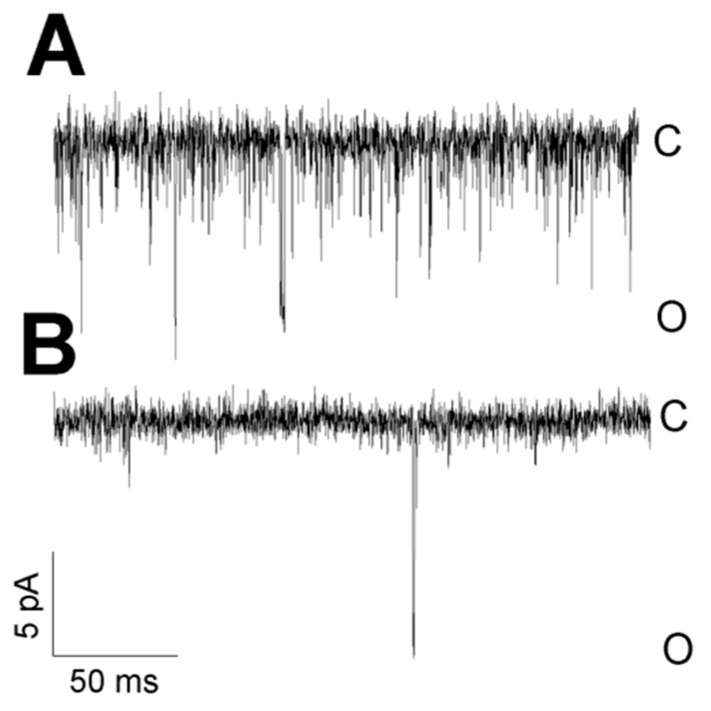
The flickering mode depends on cytosolic Ca^2+^. (**A**) High-frequency flickering was observed in cells treated with thapsigargin in the cell-attached configuration. Thapsigargin enhances the concentration of cytosolic Ca^2+^, and 1.7 mM Ca^2+^ was present in the bath solution. (**B**) TREK-2-like channels in cells treated with BAPTA-AM had no flickering kinetics in the cell-attached configuration. BAPTA-AM removes Ca^2+^ from the cytoplasm, and 0.1 mM Ca^2+^ was present in the bath solution. Both traces were recorded at −50 mV.

Two modes of activity were characteristic for TREK-2-like channels. The TREK-2-like channel activity occurred either as bursts of well-resolved openings or as flickering activity with very fast closure kinetics. The two modes of activity had different voltage dependences: while well-resolved bursts of long openings were very often observed during depolarization, flickering was promoted by hyperpolarization (Figure 10). Flickering often preceded the loss of activity. However, strong hyperpolarization facilitated the transition from the flickering state to the state of typical activity (Figure 2A: −75 mV). With increased hyperpolarization, the frequency of the flickering increased in each studied patch (Figure 10B). The flickering state depended on the level of cytosolic Ca^2+^ (Figure 11). In the absence of Ca^2+^ secured by BAPTA-AM, no flickering activity was present in any of the recorded patches (Figure 11B, *n* = 6). In contrast, with thapsigargin in the bath solution, the flickering was predominant (Figure 11A, *n*= 15). 

## 4. Discussion

The main goal of this study was to understand how TREK-2-like channels in mPFC pyramidal neurons activate spontaneously in the absence of exogenous stimuli. We reasoned that the activity of the high-conductance potassium channel, preferentially representing the activity of a non-canonical splice variant of the TREK-2 channel [36], occurs at a substantial expense of the cell’s energy and thus needs to be strictly regulated. All TREK channels are subject to complex regulation. The activation mechanisms of TREK channels are numerous and include mechanical, thermal, and chemical stimulation [18]. The mechanisms of TREK channel activation suggest that they play a neuroprotective role in ischemia of the central nervous system [55,56]. In such conditions, the stimuli activating TREK channels accumulate as arachidonic acid, and lysophospholipids are released in the ischemic focus, swelling occurs, and cell membranes are stretched as a consequence [20,57]. Activated by these stimuli, the TREK channels induce hyperpolarization of neuronal membranes, which contributes to a decrease in the energy demand of the neuron under unfavourable conditions. The activity of TREK channels is, therefore, an important element of neuronal bioenergetics.

Our research shows that the activity of TREK-2-like channels is dependent on cytoplasmic Ca^2+^. We rejected the possibility that Ca^2+^ alone is able to activate the channel because, in the presence of the PLC activator, P_open_ decreased (Figure 5). The PLC activator diminishes the PI(4,5)P2 pool [46] and increases the cytoplasmic Ca^2+^ concentration, but the Ca^2+^ increase was insufficient to maintain the channel activity. TREK-2 channels are dependent on PI(4,5)P2; we therefore focused on the mechanism of TREK-2-like channel activation based on its interaction with PI(4,5)P2 [29,32,33,40]. In particular, we considered possible mechanisms involving both Ca^2+^ and PI(4,5)P2. When considering such mechanisms, it should be noted that multivalent cations, including Ca^2+^, interact with PI(4,5)P2, allowing them to self-aggregate. This leads to the formation of negatively charged domains, nanometer-scale PI(4,5)P2 clusters, where PI(4,5)P2 molecules are bridged by calcium ions. Calcium-induced PI(4,5)P2 clustering was studied by FRET, atomic force, and electron microscopy as well as by infrared spectroscopy and other methods [58,59,60]. 

**Figure 12:**
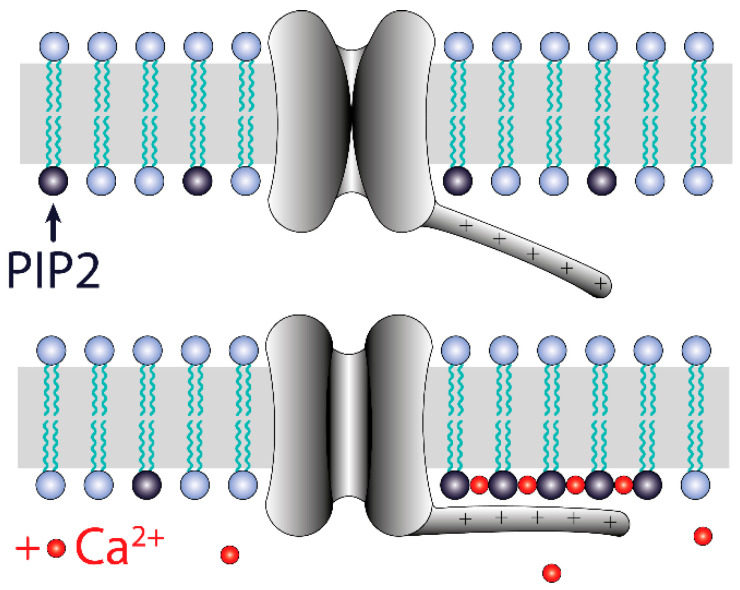
Model of TREK-2-like channel activation by the PI(4,5)P2/Ca^2+^ nanocluster. Ca^2+^ facilitates the aggregation of PI(4,5)P2 molecules in the inner leaflet of the plasma membrane. Calcium ions are attracted to the plasma membrane during depolarization, so the formation of PI(4,5)P2 nanoclusters is more likely at positive potentials than at negative potentials. The nanoclusters electrostatically tether the C-terminus domain bearing positively charged amino acids. This interaction stabilizes the open-channel conformation. The voltage-dependent formation of nanoclusters results in voltage dependency of the TREK-2 channel activity.

Our results indicate that PI(4,5)P2-dependent TREK-2 activity occurs when there are conditions for PI(4,5)P2/Ca^2+^ nanocluster formation: TREK-2-like channel activity significantly increased when the presence of PI(4,5)P2 and Ca^2+^ was secured (Figure 9) and decreased in the presence of a PLC activator, polycations (such as neomycin), and BAPTA-AM (Figure 4 and Figure 11B). We, therefore, speculate that the mechanism of calcium action is to enable the formation of PIP2 nanoclusters and that such calcium-induced nanoclusters facilitate the interaction of the TREK-2-like channel with the membrane, which results in channel activation (Figure 12). Our finding that 100 μM Ca^2+^ is sufficient to induce high activity in TREK-2-like channels (Figure 8) is important in view of the observation that, at Ca^2+^ levels of 100 μM, PI(4,5)P2 is highly clustered [58]. Many channels appear to be both activated and inhibited by PI(4,5)P2, including TREK-1, TRPV1, K_v_, HCN, Ca_v_, and TRP [61]. However, to our knowledge, the involvement of PI(4,5)P2/Ca^2+^ nanoclusters in the activation of ion channels has not been demonstrated so far; therefore, our research suggests a novel pathway for channel activation, which should, however, be confirmed by more detailed research. The observation that calcium participates in the activation of TREK-2-like channels is a new finding. 

Ca^2+^ is an important intracellular second messenger involved in many signalling pathways. Below, we explain why we rejected other possible mechanisms of calcium action on the channel. Possible pathways for calcium interaction with ion channels include DAG kinase and PKC. Both kinases have been shown to be Ca-dependent [62,63]. The phosphorylation of DAG by DAG kinase leads to the production of phosphatidic acid, which is an activator of TREK channels. However, this pathway, while possible in cell-attached experiments, is unlikely in inside-out experiments. We also reject the possibility that Ca^2+^ acts via PKC activation because TREK-2 phosphorylation results in channel inhibition, which is in contrast with our experiments in which calcium presence resulted in increased channel activity. 

The experiments showing that exogenous PI(4,5)P2 increases the activity of TREK-2 channels [26] could suggest that Ca^2+^ acts via the stimulation of PI(4,5)P2 synthesis. The increase in the PI(4,5)P2 pool was observed after the stimulation of G_q/11_-coupled muscarinic receptors [64]. The synthesis of PI(4,5)P2 involves two phosphatidylinositol kinases: (PI) 4-kinase and (PIP) 5-kinase. Ca^2+^ stimulates (PI) 4-kinase indirectly via calmodulin-like neuronal calcium sensor 1 [65]. We cannot exclude that the increase in the PI(4,5)P2 pool by elevated cytoplasmic calcium could contribute to the observed increase in TREK-2-like channel activity in the experiments performed in a cell-attached configuration. However, we exclude such a possibility in inside-out experiments due to the fact that they were performed in the absence of ATP, which is a substrate for PI phosphorylation.

The calcium dependence of TREK-2 channel activity has not yet been reported in published studies. The effect of calcium on TREK-2 channels has been investigated in heterologous expressing systems using CHO cells, and those authors did not observe any significant effect of Ca^2+^ in inside-out patches [66]. In contrast with these data, our research concerns the high-conductance channel, possibly representing the non-canonical splice variant of TREK-2, with a conductance much greater than that of the canonical isoform described in cited research [5]. Whether the effect of calcium on TREK-2-like channels is a specific feature of pyramidal neurons of mPFC or is a common feature of other cells requires further research, in particular, whether an isoform of the channel is crucial needs to be clarified.

Pyramidal mPFC neurons express the classic high-conductance potassium channel dependent on calcium (K_BK_) [67]. To eliminate the possibility that the observed activity involves K_BK_ channels, two blockers of these channels were used simultaneously (charybdotoxin or paxilline and penitrem A). Additionally, TREK-2-like channels and K_BK_ channels have distinct kinetics. K_BK_ channels are strongly voltage-dependent, and even at high calcium concentrations, a large difference in activity between the hyperpolarized and depolarized potentials is present [68]. TREK-2-like channels are weakly voltage-dependent, and their activity at hyperpolarized potentials is often of similar intensity as at depolarized potentials (Figure 2).

The gating of TREK-2-like channels was polymodal. The activity of TREK-2-like channels occurred either as bursts of well-defined openings and closings or as rapid openings, the opening times of which were at the limit of the apparatus resolution; hence, some openings had reduced amplitudes (Figure 10B). The activity of TREK-2-like channels in the burst mode increased with depolarization (Figure 2), whereas in the flickering mode, it increased with hyperpolarization (Figure 10B). The depolarization-induced increase in P_open_ of bursts could possibly be due to the interaction of calcium with the membrane, facilitated by depolarization. We speculate that the voltage dependence of the TREK-2-like channel activity in burst mode is due to the electrostatic attraction of Ca^2+^ to the membrane when the membrane is depolarized and thus to the voltage-dependent PI(4,5)P2/Ca^2+^ nanocluster formation. Although they do not have a canonical voltage-sensing domain, TREK channels show voltage-dependent activity [69]. The reason for this voltage dependency has been attributed to the voltage-dependent movement of K^+^ within the selectivity filter [70] or to the interaction of the C-terminus domain with the membrane [69]. Our suggestion that the voltage dependence of TREK-2 channel activity is due to the voltage dependence of nanocluster formation is a new contribution to studies on this phenomenon and agrees with the results of Maingret et al. [69]. 

Flickering (fast openings) occurred only under conditions of elevated calcium. Since flickering often preceded the loss of activity, we suggest that the flickering corresponds to a partially desensitized channel state, which precedes the state of full channel desensitization. Strong hyperpolarization (usually −75 mV) facilitated the transition from the flickering state to the state of typical activity (bursts,), suggesting that a partially desensitized channel can recover from desensitization during hyperpolarization. Desensitization of TREK-2 channels has not been previously reported. However, the PI(4,5)P2-induced inhibition of TREK-2 channels observed at elevated PI(4,5)P2 concentrations could possibly be another manifestation of channel desensitization [38].

Calcium-induced activation of TREK-2-like channels may be physiologically important in pathophysiological transient cellular hypercalcemia. It may reveal a novel calcium-dependent mechanism that prevents the neuron from becoming deleteriously overactive, e.g., it could prevent epileptiform activity [71,72] or activity in other pathological conditions

## 5. Conclusions

mPFC pyramidal neurons express high-conductance potassium channels with the characteristics of two-pore domain TREK-2 channels. These channels are regulated by various physical and chemical factors. Our research indicates that cytoplasmic calcium is an important factor for increasing the channel activity and that this effect is enhanced in the presence of PI(4,5)P2.

## Data Availability

The data presented in this study are available from the corresponding author upon request.
